# The Recombinase Polymerase Amplification Test for *Strongyloides stercoralis* Is More Sensitive than Microscopy and Real-Time PCR in High-Risk Communities of Cusco, Peru

**DOI:** 10.3390/pathogens13100869

**Published:** 2024-10-03

**Authors:** Jose L. Malaga, Martha V. Fernandez-Baca, Alejandro Castellanos-Gonzalez, Melinda B. Tanabe, Clara Tift, Maria Luisa Morales, Martha Lopez, Angela Valdivia-Rodriguez, Frecia Mamani-Licona, Miguel M. Cabada

**Affiliations:** 1 Sede Cusco—Instituto de Medicina Tropical Alexander von Humboldt, Universidad Peruana Cayetano Heredia, Lima 15102, Peru; jmalagag@ucsm.edu.pe (J.L.M.); vanessa.fbc.4@gmail.com (M.V.F.-B.); malu.morales.fernandez@upch.pe (M.L.M.); martlop2000@gmail.com (M.L.); amariavalrod@hotmail.com (A.V.-R.); frecia95@hotmail.com (F.M.-L.); 2 Universidad Peruana Cayetano Heredia—University of Texas Medical Branch Collaborative Research Center—Cusco, Universidad Peruana Cayetano Heredia, Cusco 08002, Peru; 3 Division of Infectious Diseases, Department of Internal Medicine, University of Texas Medical Branch, Galveston, TX 77555, USA; alcastel@utmb.edu (A.C.-G.); mbtanabe@utmb.edu (M.B.T.); 4 Department of Internal Medicine, University of Texas Medical Branch, Galveston, TX 77555, USA; cttift@utmb.edu

**Keywords:** *Strongyloides stercoralis*, microscopy, PCR, isothermal PCR

## Abstract

Strongyloidiasis is a neglected, soil-transmitted helminth infection prevalent worldwide. The true burden of strongyloidiasis is unclear due to the lack of sensitive, field-friendly diagnostic tests. PCR tests to detect *Strongyloides* DNA in stool are sensitive and specific, but the need for expensive equipment limits their use in endemic regions. Isothermal PCR amplification tests are easier to deploy while maintaining sensitivity and specificity. We developed and evaluated a recombinase polymerase amplification lateral flow assay (RPA-LFA) to detect *Strongyloides stercoralis* in human stool samples. Three hundred stool samples were collected in three communities in the jungle of Cusco, Peru. Samples were tested for *S. stercoralis* larvae using microscopy (Baermann’s, agar plate culture (APC), and rapid sedimentation), real-time PCR, and RPA-LF for *Strongyloides* DNA. The RPA-LFA showed an analytical limit of detection of 20 pg/µL. The prevalence of *S. stercoralis* was 27%, 38%, 46.3%, and 46% using microscopy, PCR, microscopy/PCR, and RPA-LFA, respectively. RPA-LFA had a sensitivity and specificity of 59.3% and 58.9%, 66.2% and 71.4%, and 77.2% and 73.1% when microscopy, microscopy/PCR, and real-time PCR were used as the gold standards, respectively. The *Strongyloides* RPA-LFA is a novel, fast, highly sensitive, and specific molecular method with the potential for deployment in endemic regions.

## 1. Introduction

Strongyloidiasis, caused by the soil-transmitted helminth (STH) *Strongyloides stercoralis*, is a neglected tropical disease [1]. Patients with strongyloidiasis can have non-specific symptoms such as chronic abdominal pain, diarrhea, or weight loss. Autoinfection, which occurs when larvae mature within the host and cause new infections, allows *Strongyloides* to cause long-term illness. Severe and fatal hyperinfection may occur in susceptible subjects, such as those with HTLV-1 or those receiving immunosuppressive medications [2,3]. In contrast with other STH, commonly used stool microscopy techniques cannot readily detect *Strongyloides* larvae, and the infection responds poorly to mass drug administration with albendazole [4,5]. These characteristics of strongyloidiasis hinder efforts to measure the prevalence of infection, estimate disease burden, and create effective control strategies.

The first estimates of the global prevalence of *Strongyloides* infection suggested between 30 and 100 million cases [6]. However, these estimates lacked a clear methodology and were probably based on insensitive microscopy techniques [7,8]. Buonfrate et al.’s estimates were based on studies using *Strongyloides*-specific diagnostic methods (Baermann’s, agar plate culture (APC), and PCR) and studies using other methods, but adjusting the prevalence reported in the latter for the lack of sensitivity. The spatiotemporal modeling in this study estimated a global prevalence of 614 million strongyloidiasis cases [3]. Fleitas et al. estimated that 386 million people worldwide have strongyloidiasis, based on biological and epidemiologic similarities between hookworm and *Strongyloides* [9]. The large differences between estimates call for better diagnostic tools for use in endemic areas. An accurate count, coupled with studies addressing strongyloidiasis morbidity, would enable burden estimations and highlight the need to control *Strongyloides* [10].

There are no gold standard tests for strongyloidiasis diagnosis. The Baermann test and APC are the most sensitive stool tests for detecting *Strongyloides* infections [11]. The Baermann test is four times more sensitive than direct smear or formalin-ether concentration techniques [12]. The APC has the same sensitivity as the direct smear, formalin-ether concentration, and the Harada–Mori test combined [7]. Comparisons between the Baermann test and APC have yielded inconsistent results. A meta-analysis of 11 studies including 9000 subjects showed that the Baermann test had a sensitivity of 72% compared to 89% for APC [13]. In contrast, a recent study comparing different microscopy techniques suggested that the sensitivity of Baermann test outperformed that of APC (*p* = 0.03) but was not better than that of sedimentation [14]. Modifications to the Baermann test, such as the addition of a charcoal pre-incubation step, can increase the sensitivity of the test compared to the original version [15]. The detection of IgG antibodies is highly sensitive in immunocompetent patients compared to stool testing [16]. However, serology has a lower sensitivity (43%) in immunosuppressed patients [17]. Other drawbacks of antibody detection for strongyloidiasis in endemic areas include cross-reactivity with other helminths and the persistence of antibodies in the absence of active infection [18,19]. 

DNA amplification tests by conventional PCR and isothermal techniques have been used to diagnose *Strongyloides* infection in humans [20,21,22]. The sensitivity of real-time PCR varies widely between studies. Verweij et al., using a real-time PCR targeting the *S. stercoralis* 18s rRNA gene, showed a sensitivity of 91% and specificity of 92% using Baermann and APC tests as the reference tests [20]. Hailu et al., using the same real-time PCR, reported a sensitivity of 74% using the combination of formol-ether concentration, spontaneous in-tube sedimentation, Baermann, and APC as the reference tests. In this study, the agreement between the real-time PCR and each stool microscopy technique was only slight (kappa statistic, 0.025–0.032) [21]. Kristanti et al. reported a lack of agreement between a PCR targeting the internal transcribed spacer 2 of the rDNA gene of *S. stercoralis* and the Baermann and Koga APC tests. In this study, subjects with a low number of parasites in their stool were less likely to test positive on the three tests [22]. A recent meta-analysis evaluating the accuracy of molecular tests for strongyloidiasis diagnosis reported a sensitivity between 64 and 72% for molecular tests using a composite of stool parasitological techniques as the reference tests. The sensitivity of the molecular tests was lower (56–62%) when serology was added to the composite reference [19]. The authors suggested that the sensitivity of molecular tests decreases in low-intensity infections. The drop in sensitivity when serology was introduced as one of the reference tests may be associated with the lack of specificity of this technique. Tamarozzi et al. compared the accuracy of Baermann test, real-time PCR, and three serologic tests including a rapid diagnostic test (RDT) using a recombinant antigen to diagnose strongyloidiasis among children in rural Ecuador [20,23]. In this study, the sensitivity and specificity of PCR were 56% and 99%, respectively. The sensitivity of the combination of PCR and RDT was over 90% [23].

However, combining an RDT with a complex equipment-dependent PCR is unlikely to impact the diagnosis in endemic areas. Isothermal amplification methods may overcome these issues as they require little equipment and infrastructure [24,25,26]. We report on the development of an isothermal recombinase polymerase amplification test targeting the *Strongyloides* 18s rRNA gene in human stool and compare it to real-time PCR and *Strongyloides* stool microscopy tests using a community-based sample of subjects in a highly endemic area of Peru.

## 2. Materials and Methods

### 2.1. Sample Procurement and Processing

Stool samples were collected in the Quellouno district (population 18,089), located in the province of La Convencion in the Cusco region of Peru, at an elevation of 2600 ft in the rainforest. In collaboration with the Ministry of Health and community authorities, male and female subjects aged 3 years or older from the Putucusi, Alto Putucusi, and San Martin communities were invited to participate in an epidemiologic study [27]. Those who consented to participate received a stool collection kit and instructions to collect one stool sample early in the morning of the pre-arranged household visit. Field workers collected the samples from each household early in the morning. The stool was transported immediately to an on-site microscopy laboratory set up for the study in the local health center. Samples were separated in aliquots and used fresh for the Baermann test and APC for *S. stercoralis* detection, as described previously [7,28]. Three-gram aliquots of each stool sample were preserved in 10% formalin to perform a rapid sedimentation test or in 70% alcohol for DNA extraction at the Cusco Branch of the Alexander von Humboldt Tropical Medicine Institute of Universidad Peruana Cayetano Heredia in Cusco city.

### 2.2. DNA Extraction

DNA extraction was performed using the commercially available E.Z.N.A.^®^ Stool DNA kit (Omega Bio-Tek, Norcross, GA, USA). The alcohol-preserved samples were centrifuged, and approximately 200 mg of stool was obtained from each tube. The lysis process was modified to ensure adequate parasite DNA extraction. In brief, samples were combined with 200 mg of glass beads and SLX-Mlus buffer, provided by the manufacturer, and vortexed for 10 min. DS buffer and proteinase K were then added, and the specimens were incubated at 70 °C for 10 min. Then, the specimens underwent three 20 min cycles of freezing (−80 °C) and heating (95 °C). Finally, we used the lysate for DNA extraction according to the manufacturer’s instructions. We determined the DNA quality and concentration by spectrophotometry using a NanoDrop 2000 spectrophotometer (Thermo Scientific, Wilmington, DE, USA).

### 2.3. Real-Time PCR for Strongyloides stercoralis

We adapted a previously published primer set targeting the *S. stercoralis* 18s rRNA gene (GenBank MN607960.1 and EF653266.1) for real-time PCR using SYBR Green-based chemistry [29] (Table S1). The final reaction included 10 µL of Sso Advanced Universal SYBR Green Supermix (BioRad, Hercules, CA, USA), 1 µL of forward primer (10 µM), 1 µL of reverse primer (10 µM), 2 µL of DNA template (concentration range 20–80 ng/µL), and 6 µL of nuclease-free water. The experiments were performed using a CFX-96 Touch Real-Time PCR System (BioRad, Hercules, CA, USA) under the following cycling conditions: 50 °C for 5 min, 98 °C for 2 min, (98 °C for 10 s, 58 °C for 45 s, 72 °C for 45 s) for 35 cycles, followed by melting curve analysis. Duplicate reactions were performed for each DNA sample and the controls. We included a *S. stercoralis* DNA positive control and a nuclease-free water no-template negative control in all real-time PCR experiments. Samples were considered positive for *S. stercoralis* if both reactions showed similar amplification curves, the products had the same melting point (±1 °C) as the positive control, and the non-template control showed no amplification. Samples were considered negative if no amplification curves were detected, the positive controls showed amplification at the expected melting point, and the negative controls did not show amplification. Samples that did not meet these definitions were considered indeterminate and were tested again.

### 2.4. RPA for Strongyloides stercoralis

We designed the primer set for the RPA test targeting the same region of the *S. stercoralis* 18s rRNA gene (GenBank MN607960.1 and EF653266.1) used for the real-time PCR [26,29]. The resulting primers were 4–5 nucleotides longer than the ones used for the PCR. The *Strongyloides* RPA for lateral flow (LFA) detection was standardized using the commercially available TwistAmp nfo kit (TwistDX, Cambridge, UK) with a 5′ FAM (5′ carboxy fluorescein amidite)-labeled *Strongyloides* probe containing an internal spacer C3 in 3′. The reverse primer was labeled with biotin at the 5′ end for LFA (Table S1). A positive RPA reaction generated a double-labeled product with biotin from the reverse primer and FAM from the *Strongyloides* probe. The reaction volume was 50 µL and included the TwistAmp nfo dried enzymes, 29.5 µL of rehydration buffer supplied with the kit, 1.5 µL of forward primer (10 µM), 1.5 µL of biotinylated reverse primer (10 µM), 0.8 µL of FAM-labeled probe (10 µM), 11.7 µL of nuclease-free water, 2 µL of DNA template (same concentration range), and 3 µL of 280 mM magnesium acetate. The reaction temperature and incubation time were adjusted to optimize the detection of the target sequence. The final reaction conditions involved incubation at 38 °C for 30 min in an analog heat block. Agarose gel electrophoresis was used to visualize the RPA-nfo amplicons and to evaluate the limit of detection (LOD) and sensitivity. We used a 2% agarose gel stained with ethidium bromide and a 100 bp DNA ladder to determine the predicted product size.

### 2.5. Lateral Flow Detection (RPA-LFA)

To detect the double-labeled RPA products using lateral flow strips, 2 µL of the RPA-nfo amplification reaction were diluted in 98 µL of phosphate-buffered saline (PBS) with Tween solution in a separate 1.5 mL tube. A commercially available lateral flow strip (Ustar Biotechnologies, Hangzhou, China) was immersed in the solution and allowed to soak for 10 min before reading. The strips contained carbon nanoparticles conjugated with anti-FAM antibodies that bound to the double-labeled RPA products as they flow to the test line by capillarity. The anti-biotin antibodies in the test line bound to the biotin in the products and immobilized them, generating a visible band. The reactions were considered positive if the test line showed a band and the control line showed a band formed by excess unbound gold nanoparticles immobilized by anti-antibodies. The reactions were considered negative if only the control line showed a band. All RPA-LFA experiments contained an *S. stercoralis* DNA positive control and a no-template negative control.

### 2.6. Limit of Detection and Analytic Specificity of PCR and RPA-LFA

The LOD for the *S. stercoralis* real-time PCR and RPA was tested using 10-fold dilutions of DNA extracted from *S. stercoralis* L1 larvae enriched from stool by the Baermann test and washed in PBS. The LOD for the RPA was initially evaluated by agarose gel electrophoresis, and once optimized, it was evaluated using lateral flow strips (RPA-LFA) to ensure that the test maintained the same LOD. DNA from stool samples containing *Dipylidium caninum*, *Trichuris trichiura*, *Ascaris lumbricoides*, and hookworm, as well as DNA from *Fasciola hepatica*, *Taenia solium*, and human specimens, was extracted using the same method as described above and used to test the analytical specificity of the *Strongyloides* real-time PCR and RPA reactions. The stool samples for specificity testing were retrieved from a biorepository containing microscopy-characterized specimens collected at 3400 m above sea level from subjects that tested negative for *Strongyloides* in three stool specimens. The prevalence of *S. stercoralis* in that population was 1.4% [30].

### 2.7. Clinical Sensitivity and Specificity of RPA-LFA

The sensitivity and specificity of the *Strongyloides* RPA-LF were evaluated by comparing the test results with three different gold standards. The microscopy composite gold standard was defined as the combination of all microscopy test results (Baermann test, APC, and rapid sedimentation test). The PCR gold standard was defined by the qualitative results of the real-time PCR test. The microscopy–PCR gold standard was defined by the combination of the microscopy and PCR gold standards. The results of the microscopy tests, real-time PCR, and RPA-LFA were blinded to the laboratory personnel performing the tests.

### 2.8. Data Analysis

The Statistical Package for the Social Sciences version 25.0 (SPSS, Inc., Chicago, IL, USA) was used for the analysis. A *p* < 0.05 was considered statistically significant using two-tailed values. The sensitivity, specificity, and their 95% confidence intervals were calculated. The McNemar test was used to compare test sensitivities. The kappa statistic was used to evaluate the agreement between tests. The kappa statistic values were classified as follows: poor agreement (kappa < 0.00; 0.00–0.20), slight agreement (kappa = 0.21–0.40), fair agreement (kappa = 0.41–0.60), moderate agreement (kappa = 0.61–0.80), and substantial agreement (kappa = 0.81–1.00).

### 2.9. Ethical Statement

The study protocol was approved by the Universidad Peruana Cayetano Heredia Institutional Ethics Committee (#64250). Subjects who agreed to participate voluntarily provided written informed consent before enrollment in the study. Children provided assent for participation and their parents provided informed consent.

## 3. Results

### 3.1. Analytical Characteristics of Strongyloides Real-Time PCR and RPA

**Figure 1:**
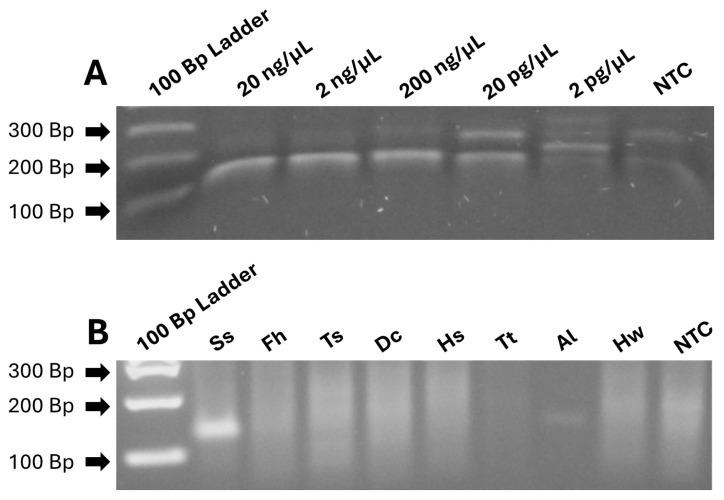
Characteristics of the *Strongyloides stercoralis* RPA evaluated in 2% agarose gel electrophoresis stained with ethidium bromide. Panel (**A**): Limit of detection using *Strongyloides* DNA from parasites enriched by the Baermann test. Panel (**B**): Cross-reactivity evaluated using specimens containing DNA from *Strongyloides stercoralis* (Ss), *Fasciola hepatica* (Fh), *Taenia solium* (Ts), *Dipylidium caninum* (Dc), Human (Hs), *Trichuris trichiura* (Tt), *Ascaris lumbricoides* (Al), and hookworm (Hw). NTC = nuclease-free water.

The real-time PCR and the RPA successfully amplified the 115 bp *Strongyloides* 18s rDNA gene target. The limit of detection for both the real-time PCR and RPA-LFA was 20 pg/µL of *S. stercoralis* DNA, equivalent to 17.4 copies/reaction, as calculated according to methods described elsewhere [31] (Figure 1A). The real-time PCR and RPA of samples containing DNA of *Fasciola hepatica*, *Taenia solium*, *Dipyllidium caninum*, *Trichuris trichiura*, and hookworm showed no cross-reactivity. The agarose gel electrophoresis of the RPA products showed a weak band of higher molecular weight with DNA of a stool sample positive for *Ascaris lumbricoides*, but the real-time PCR did not show an amplification curve. The purification of RPA products and the denaturation of the nucleoproteins that form the RPA recombinase primer complex with 2-mercaptoethanol made the unspecific band disappear from gel electrophoresis (Supplementary Figure S1). Neither the real-time PCR nor the RPA test showed amplification with samples containing human DNA (Figure 1B).

**Figure 2:**
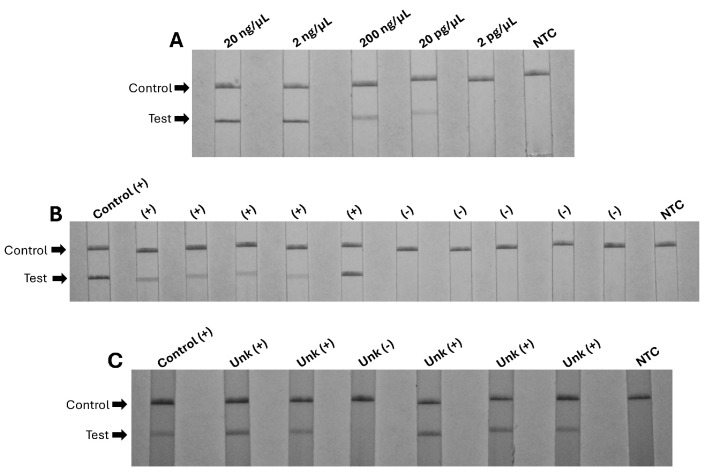
Evaluation of the RPA using lateral flow strips to detect the reaction products. Panel (**A**): Limit of detection of 20 pg/μL of *Strongyloides* DNA from parasites enriched by the Baermann test. Panel (**B**): RPA LFA using 5 banked stool specimens with positive microscopy (+) and 5 specimens with negative microscopy (−). Panel (**C**): RPA LFA results in the clinical samples (Unk) collected for the study. The (+) positive control used *Strongyloides* DNA extracted from Baermann-enriched parasites. The no-template control (NTC) used nuclease-free water.

The RPA adapted for lateral flow strips maintained the same limit of detection of 20 pg/μL and was able to detect *Strongyloides* DNA in banked stool samples with known positive microscopy. The RPA-LFA did not show cross-reactivity with human DNA or DNA from *Fasciola hepatica*, *Taenia solium*, *Dipyllidium caninum*, *Trichuris trichiura*, hookworm, or *Ascaris lumbricoides* (Figure 2).

### 3.2. Testing of Clinical Samples

**Figure 3:**
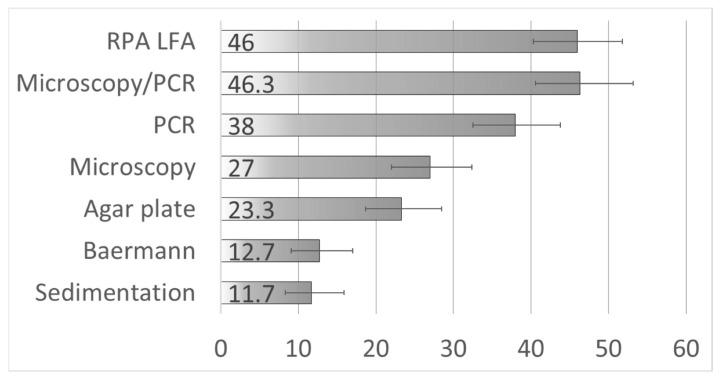
Prevalence of *Strongyloides stercoralis* among participants, estimated using different tests (N = 300). The bars depict the percentage and error bars the 95% confidence interval.

Three hundred and forty-nine participants from the communities of San Martin, Alto Putucusi, and Putucusi were enrolled in the study and provided one stool sample each. After excluding subjects whose stool samples were not enough to be tested by all five methods, 300 subjects were included in the evaluation of the *Strongyloides* RPA-LFA characteristics. The prevalence of *Strongyloides*, combining the results of all microscopy tests and real-time PCR, was 46.3% (95% CI: 40.6–52.2). The rapid sedimentation test estimated the lowest prevalence (11.7% (95% CI: 8.3–15.9)) and the real-time PCR (38% (95% CI: 32.5–43.8)) estimated the highest. The prevalence of strongyloidiasis estimated by RPA-LFA was 46% (95% CI: 40.3–51.8) (Figure 3). In total, 1 sample (1/35) was positive only by the rapid sedimentation test, 1 sample (1/38) only by Baermann’s test, 14 samples (14/114) only by PCR, 17 samples (17/70) only by APC, and 46 samples (46/138) only by RPA-LFA. Twenty samples were positive by all five tests.

The sensitivity and specificity of the *Strongyloides* RPA-LFA were 59.3% (95% CI: 49.4–68.6) and 58.9% (95% CI: 55.2–62.4), respectively, when compared with the combined results of the microscopy tests. When compared to the real-time PCR, the sensitivity and specificity were 77.2% (95% CI: 70.1–83.3) and 73.1% (95% CI: 68.8–76.8). The kappa statistic showed poor agreement with microscopy (kappa = 0.14) and a fair agreement with the real-time PCR (kappa = 0.48). The McNemar Chi-square test showed significant differences between the RPA-LFA and both the microscopy composite and the real-time PCR (Table 1).

## 4. Discussion

The diagnosis of strongyloidiasis in the community is challenging due to the poor performance of the diagnostic tests available. This is explained in part by the low number and intermittent excretion of larvae in the stool [32]. Molecular diagnosis allows for the detection of active *Strongyloides* infection with high sensitivity and specificity [33]. We developed an RPA-LFA test to detect *S. stercoralis* DNA in stool samples that outperformed microscopy and real-time PCR. The RPA-LFA performance varied significantly depending on the standard to which the test was compared. The sensitivity, specificity, and agreement were highest when compared with the *Strongyloides* real-time PCR. The RPA-LFA detected more strongyloidiasis cases than the real-time PCR targeting the same gene as the primers published by Moghaddassani [29]. Although a comparison with other published real-time PCR tests was not performed in our study, adapting primers from better-performing PCR tests to RPA could generate better results. The RPA-LFA has the potential to overcome common issues with molecular tests, such as the need for equipment and steady power, and may be deployed in low-complexity healthcare settings.

In our study, the *Strongyloides* RPA-LFA had a low analytical LOD (20 pg/µL) and detected more cases of strongyloidiasis than the combination of microscopy tests or real-time PCR. The LOD of RPA was comparable to other molecular tests, such as our real-time PCR. Fernandez-Soto et al. reported that the Strong-LAMP, another isothermal PCR method, had a comparable LOD of 10 pg/µL [34]. Similarly, Crego-Vicente et al., using the same Strong-LAMP test on human stool samples collected in the community, detected a significantly higher number of strongyloidiasis cases compared to the combination of microscopy tests [35]. This suggests important advantages of implementing isothermal PCR testing over microscopy. We have reported before that RPA outperforms real-time PCR when testing stool samples for *Fasciola hepatica*, detecting a higher number of cases in microscopy false-negative samples [25]. Our RPA-LFA targets the same genomic region and has the same analytical LOD as our real-time PCR. However, a possible explanation for the better performance of RPA may be associated with its resistance to PCR inhibitors present in stool samples [36]. In addition, together, the RPA-LFA and microscopy composite detected the highest number of cases compared to any other combination of tests. Combining different methods may improve the sensitivity of strongyloidiasis diagnosis [23]. Combinations of isothermal amplification tests with microscopy may be suitable for endemic areas where equipment and reliable power are lacking.

The agreement of RPA-LFA with the combined microscopy tests was poor. This has been reported in other diagnostic studies in strongyloidiasis. Chankongsin et al. evaluated strongyloidiasis patients admitted to the hospital for unrelated problems in the Lao People’s Democratic Republic. The authors used wet mount, Baermann, Koga APC, and real-time PCR to test for *Strongyloides* infection in the stool [37]. Only a third of the subjects with strongyloidiasis tested positive by all four methods, and the agreement between the microscopy tests and the real-time PCR was moderate [37]. Similarly, Kristanti et al. evaluated the agreement of a PCR test for *Strongyloides*, testing stool samples that were positive or negative by the Baermann test and/or Koga APC. The agreement between testing methods was highest (75%) when both the Baermann test and Koga APC were positive and lowest (45%) when only the Koga APC test was positive. When both the Baermann test and the Koga APC were negative, the PCR was positive in 8 out of 20 samples [22]. A low burden of infection and an uneven distribution of larvae in the stool may partially explain these differences. The amount of stool we used for DNA extraction (200 mg) with a commercial kit was low and may have missed larvae that could otherwise be detected by microscopy testing a few grams of stool. In addition, we studied a population of asymptomatic individuals in the community who may have low-intensity infections. Microscopy has an even lower sensitivity in low-intensity infections, explaining the poor agreement between microscopy and PCR tests [22,38]. PCR-based tests have better sensitivity than microscopy in endemic areas, despite the lack of agreement with other tests [19,39,40].

The interpretation of the results of our study must take into consideration some limitations. This was an exploratory study that used stool samples collected from subjects in high-risk communities in the jungle of Cusco. Although the participants were asymptomatic, the prevalence of infection was probably high. The performance of the RPA-LFA in areas of low prevalence might be different and needs further investigation. In addition, the RPA-LFA should be optimized by testing other primer sets and conditions before implementation. We did not include serology as one of the comparators which, in a highly endemic area such as Quellouno, could have detected a larger number of subjects exposed to *Strongyloides*. However, some of the commercially available tests may not be able to differentiate between active infection and recent exposure and could cross-react with other helminth infections. *Strongyloides* serology is not dependent on the number of larvae in the stool, and newer tests have shown better specificity, which could have provided important information to help differentiate false-positive and false-negative results in our study. In addition, no internal DNA controls were included in the PCR or RPA reactions, which further limited the interpretation of negative results. A limiting factor for the deployment of the RPA-LFA is the need for the manipulation of the amplification products after the reaction. In settings where training and facilities are suboptimal, such product manipulation may cause contamination of reagents, false-positive results, and a waste of resources. Our methods included controls and a rigorous definition to validate each run and detect potential false positives and negative results. Adapting the RPA reaction to a miniature device, where DNA extraction, amplification, and product detection occur, may eliminate the need for post-reaction manipulation.

In conclusion, the RPA-LFA for detecting *S. stercoralis* in human stool was highly sensitive and outperformed microscopy and RT-PCR tests. The RPA-LFA is a promising method for a sensitive and specific field diagnosis of strongyloidiasis. However, further research should focus on DNA extraction methods paired with closed amplification product detection systems to limit contamination and false-positive risk.

## Figures and Tables

**Table 1:** Sensitivity, specificity, kappa statistics, and McNemar’s test between the SS RPA-LFA and other tests for diagnosis of strongyloidiasis.

	Microscopy		Microscopy/PCR		PCR	
	Positive	Negative	Positive	Negative	Positive	Negative
RPA LF Positive	48	90	92	46	88	50
RPA LF Negative	33	129	47	115	26	136
Sensitivity *	59.3 (49.4–68.6)		66.2 (60–71.9)		77.2 (70.1–83.3)	
Specificity *	58.9 (55.2–62.4)		71.4 (66–76.3)		73.1 (68.8–76.8)	
Kappa *	0.149 (0.038–0.254)		0.376 (0.26–0.482)		0.483 (37.4–57.8)	
McNemar	<0.001		1		0.008	

* Value (95% confidence interval).

## Data Availability

The datasets generated and analyzed during the current study are available from the corresponding author upon reasonable request.

## Supplementary Materials

The following supporting information can be downloaded at https://www.mdpi.com/article/10.3390/pathogens13100869/s1: Table S1: Primer and probes sequences for *S. stercoralis* PCR and RPA LF; Figure S1: Cross-reactivity test.
