# Supplementary material for: The Recombinase Polymerase Amplification Test for Strongyloides stercoralis Is More Sensitive than Microscopy and Real-Time PCR in High-Risk Communities of Cusco, Peru
Source: Pathogens. 2024 Oct 3;13(10):869. doi: 10.3390/pathogens13100869 (PMC11510723; doi:10.3390/pathogens13100869)
Supplement: Supplementary file 1 [file pathogens-13-00869-s001.zip › pathogens-3208306-supplementary.pdf]

Table S1. Primer and probes sequences for *S. stercoralis* PCR and RPA LF

	Primer or Probe	Sequence
PCR	Forward	3'- ATC GTG TCG GTG GAT CAT TC -5'
	Reverse	5'- CTA TTA GCG CCA TTT GCA TTC -3'
RPA	Forward	3'- ATC GTG TCG GTG GAT CAT TCG GTT -5'
	Reverse	5'- /Biotin/CTA TTA GCG CCA TTT GCA TTC AAG AA -3'
	Probe	FAM/CTG CTT AGC AAT TCG CCT TAC TTA TCG CAG [THF] TTG CTG CGC TCT TCA TCG A/C3

FAM: Fluorescein dye THF: Tetrahydrofuran

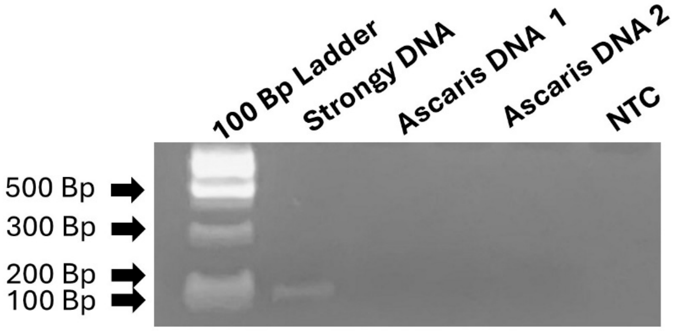

Supplementary Figure S1. Cross-reactivity test. No amplification products were detected testing two *Ascaris lumbricoides* DNA samples. *Ascaris* DNA 1: DNA sample isolated from *Ascaris lumbricoides* parasite tissue (200 Fg/µL). *Ascaris* DNA 2: DNA sample isolated from human stool characterized positive to *Ascaris lumbricoides* 100 pg/µL. NTC: Non-template control.
